# Lightweight Deep Learning for Automated Dental Caries Screening from Pediatric Oral Photographs

**DOI:** 10.3390/diagnostics16060862

**Published:** 2026-03-13

**Authors:** Nourah Alangari, Nouf AlShenaifi

**Affiliations:** Department of Computer Science, College of Computer and Information Sciences, King Saud University, Riyadh 11543, Saudi Arabia; noalshenaifi@ksu.edu.sa

**Keywords:** dental caries detection, cavity detection, pediatric dental screening, early childhood caries, lightweight CNNs, deep learning, interpretability, Grad-CAM

## Abstract

**Background:** Early childhood caries (ECC) affects a substantial proportion of young children worldwide, and timely screening is essential for early intervention and referral. While deep learning has shown promise for automated dental diagnostics, many existing approaches rely on computationally heavy models that limit deployment in community and mobile settings. This study investigates whether compact convolutional neural networks can achieve clinically meaningful performance for screening dental caries from oral photographs. **Methods:** We curated a dataset of 435 intraoral images from children aged 3–14 years, annotated by licensed dentists, and performed patient-level stratified splitting to prevent data leakage. Three convolutional neural networks (ResNet-18, MobileNetV3-Small, and EfficientNet-B0) were fine-tuned using ImageNet-pretrained weights and comparatively evaluated for the detection of dental caries from oral photographs. Models were trained with class-weighted cross-entropy loss and evaluated on a held-out test set using sensitivity, specificity, balanced accuracy, ROC-AUC, and PR-AUC with bootstrap 95% confidence intervals. **Results:** ResNet-18 achieved the highest balanced accuracy (0.929), weighted F1-score (0.954), and perfect sensitivity (1.00), while EfficientNet-B0 achieved the strongest threshold-independent discrimination with the highest ROC-AUC (0.978) and PR-AUC (0.990). MobileNetV3-Small maintained competitive performance (ROC-AUC 0.952; PR-AUC 0.976) with substantially lower computational complexity. **Conclusions:** In addition to performance evaluation, we incorporated an interpretability analysis using Grad-CAM to examine model decision behavior. The resulting attribution maps predominantly highlighted clinically relevant tooth regions associated with caries, providing evidence that the models rely on meaningful dental features rather than background artifacts. These results demonstrate that compact, deployment-friendly architectures can achieve clinically meaningful performance for ECC detection, supporting their suitability for scalable, real-world screening applications.

## 1. Introduction

Early childhood caries (ECC) remains one of the most prevalent chronic diseases affecting children worldwide and represents a significant public health burden. Epidemiological studies have consistently reported that untreated dental caries ranks among the most common diseases globally, disproportionately affecting young children and underserved populations [1,2]. National surveys further indicate that nearly one-quarter of children exhibit untreated cavities, with higher prevalence observed in low-income communities where access to preventive dental care is limited [3,4]. Because ECC can progress rapidly and lead to pain, infection, and impaired quality of life, timely identification and referral are critical for effective management. Consequently, scalable and accessible screening strategies that can operate outside specialized dental clinics are urgently needed.

Recent advances in artificial intelligence (AI), particularly deep learning, have transformed dental image analysis by enabling automated and objective detection of carious lesions [5,6,7]. Convolutional neural networks (CNNs) have demonstrated high diagnostic performance across multiple imaging modalities, including bitewing, panoramic, and periapical radiographs. Reported accuracies frequently exceed 80% [8], and in some controlled settings, approach near-expert performance. Beyond radiographic imaging, smartphone-based photographic screening has emerged as a promising solution for teledentistry and community health programs, offering low-cost and portable data acquisition [9,10]. Studies employing architectures such as DenseNet, U-Net, YOLO variants, and EfficientNet have shown encouraging results for caries detection and segmentation, supporting the feasibility of automated screening in real-world environments [11,12].

Despite these advances, several practical challenges remain. Many existing approaches rely on computationally heavy models or large architectures that require substantial memory and processing resources, limiting their suitability for mobile devices or low-resource settings where community screening is most needed [13]. Furthermore, prior work often emphasizes maximizing accuracy without explicitly considering deployment constraints such as model size, inference latency, or interpretability [14]. In addition, relatively few studies focus specifically on early childhood caries from intraoral photographs, and rigorous evaluation practices—such as patient-level data splitting to prevent leakage, balanced metrics, and reproducible auditing—are inconsistently reported [15]. As a result, it remains unclear whether simpler, compact architectures could provide comparable clinical performance while offering substantially better efficiency and deployability.

Addressing this gap is particularly important for ECC screening scenarios, where models must operate reliably on handheld devices, integrate into mobile health workflows, and support non-specialist users. From a practical standpoint, lightweight models may enable faster inference, lower energy consumption, and easier integration into smartphone-based tools, thereby facilitating large-scale public health adoption [16,17]. However, the extent to which compact networks can achieve clinically meaningful performance comparable to heavier architectures has not been systematically investigated.

Therefore, this study asks: To what extent can compact deep learning models achieve clinically meaningful performance for automated ECC screening compared to heavier architectures? To address this question, we evaluated three convolutional neural networks—ResNet-18, MobileNetV3-Small, and EfficientNet-B0—for binary dental caries detection from pediatric oral photographs. Models are trained using transfer learning with class-balanced optimization and evaluated using clinically relevant metrics, including sensitivity, specificity, balanced accuracy, ROC-AUC, and PR-AUC with bootstrap confidence intervals. To enhance reliability and transparency, we employ patient-level stratified splitting, comprehensive reproducibility logging, and Grad-CAM visualization for interpretability.

The main contributions of this work are threefold: (1) We provide a systematic comparison of multiple convolutional neural network architectures for dental caries detection from intraoral photographs; (2) We demonstrate that the evaluated models achieve strong diagnostic performance while maintaining suitability for deployment in community and mobile screening settings; (3) We present an interpretable and reproducible evaluation framework suitable for real-world screening applications. Together, these findings support the use of compact, mobile-friendly deep learning models as practical baselines for scalable pediatric dental screening.

## 2. Related Work

The application of artificial intelligence (AI) in dental diagnostics has transformed the detection and management of dental caries, providing an objective alternative to subjective visual-tactile and radiographic examinations [18,19]. Recent systematic reviews and meta-analyses have demonstrated that deep learning models, particularly convolutional neural networks (CNNs), achieve high diagnostic accuracy, sensitivity, and specificity across various imaging modalities [8,20]. For instance, overall accuracy for caries classification has been reported to range between 68% and 99%, depending on the image type and model architecture [8].

### 2.1. Imaging Modalities and Model Performance

The performance of AI models varies significantly based on the radiographic technique employed. Research indicates that AI systems tend to be more accurate when analyzing bitewing radiographs (approximately 80% accuracy) compared to panoramic radiographs (approximately 70% accuracy) [21]. Mohammad-Rahimi et al. [8] noted that classification models reached up to 99.2% accuracy on periapical radiographs and 95.4% on bitewing images. Furthermore, specialized architectures like the U-Net have been successfully utilized for pixel-level dental structure segmentation and caries detection [22]. Representative clinical radiograph-based deep learning systems have also been investigated for automated caries diagnosis. Lee et al. [11] evaluated a transfer learning approach using a pre-trained GoogLeNet Inception-v3 CNN for detecting dental caries on periapical radiographs. The study utilized 3000 radiographic images, divided into training/validation and testing sets, and assessed diagnostic accuracy, sensitivity, specificity, and ROC-AUC. The proposed CNN achieved diagnostic accuracies of 89.0% for premolars and 88.0% for molars, with AUC values of 0.917 and 0.890, respectively. These results demonstrated that deep CNNs can provide clinically reliable performance for radiographic caries diagnosis and highlighted the feasibility of AI-assisted interpretation of periapical radiographs in routine dental practice.

### 2.2. Smartphone-Driven Diagnostics and Accessibility

A growing area of interest is the use of smartphone-based imaging for remote screening and teledentistry, especially in underserved or low-resource settings [18,23]. Systematic evaluations of AI-driven smartphone tools, utilizing models such as YOLO variants, DenseNet201, and MobileNetV3, have shown high diagnostic accuracy for cavitated lesions [18]. While these tools offer an accessible solution for community screening, their sensitivity for early or non-cavitated lesions remains a challenge compared to advanced clinical methods [18].

### 2.3. Early Childhood Caries (ECC) and Machine Learning

Specific focus has also been placed on early childhood caries (ECC) due to its high prevalence in pediatric populations [18]. Priyanka et al. [24] highlighted that machine learning (ML) algorithms, such as Support Vector Machines (SVM) and XGBoost, can achieve accuracies of 88.76% and 97%, respectively, when predicting ECC from survey and imaging data. These models leverage diverse datasets to assess environmental and genetic risk factors, enabling personalized precision in caries prediction [24].

### 2.4. Impact on Clinical Workflow and Physician Support

One of the most critical roles of AI is its capacity to serve as a “second opinion” to assist clinicians [8]. Lee et al. [22] demonstrated that when dentists refer to CNN-model predictions as a reference, their overall diagnostic performance significantly improves, particularly for detecting initial and moderate caries that are frequently missed. While AI integration can increase the false-positive rate (decreasing positive predictive value), it significantly enhances sensitivity, ensuring that fewer early lesions go untreated [22].

### 2.5. Challenges and Limitations

Despite promising results, the field faces several hurdles, including significant heterogeneity in study designs, a lack of standardized reporting (e.g., adherence to STARD-AI or CLAIM), and the need for more clinically diverse datasets to ensure generalizability [8]. Furthermore, many models show reduced sensitivity for early-stage lesions, and clinical validation through prospective, randomized trials remains limited [18]. To better contextualize the contribution of this study, Table 1 summarizes representative prior deep learning approaches for dental caries detection and compares them with the proposed method in terms of imaging modality, model architecture, and reported performance.

As shown in Table 1, most previous studies rely primarily on radiographic imaging modalities such as bitewing or panoramic X-rays or require specialized acquisition equipment. In contrast, the present study focuses on pediatric intraoral photographic screening using standard clinical photography. Despite using lightweight and deployment-oriented architectures, the proposed models achieve competitive performance while enabling practical mobile and community-level screening scenarios.

## 3. Materials and Methods

### 3.1. Data Curation

This subsection describes the dataset construction process, including data sources, pediatric eligibility screening, expert validation procedures, and final dataset composition.

The dataset was curated to include diverse tooth positions (anterior, posterior, occlusal, and lateral views) to avoid overfitting to standardized frontal perspectives and to improve generalization across varied photographic conditions. Additionally, images were sourced from multiple geographic regions (China and the United States), introducing heterogeneity in acquisition settings and patient populations.

#### 3.1.1. Data Sources and Aggregation

To construct a pediatric dental caries detection dataset, we aggregated intraoral images from five publicly available repositories. Only images corresponding to children (primary or mixed dentition) were retained. All datasets were publicly available and accessed in accordance with their respective licensing terms.

##### Teeth or Dental Image Dataset (Mendeley Data)

Dixit Chaudhary et al. [25] provide a dataset containing 9562 pediatric intraoral images (ages 1–14 years) captured under controlled clinical conditions across maxillary and mandibular views.

From this dataset, we selected:

- 127 healthy (non-caries) images
- 30 caries images

##### AAP Oral Health Image Gallery

The American Academy of Pediatrics (AAP) Oral Health Image Gallery [26] was used to extract:

- 14 pediatric caries images

Images were selected based on clear visual evidence of dental caries in pediatric cases.

##### OMNI Dataset

The OMNI dataset (Oral Imaging for Malocclusion Issues Assessments) [27] was screened for pediatric cases. From this source:

- 120 pediatric caries images were retained

##### Dental Cavity Detection Dataset (Kaggle)

The Kaggle Dental Cavity Detection Dataset [28] contains mixed adult and pediatric cases. We manually filtered the dataset to retain only pediatric samples. From this source:

- 80 pediatric caries images

##### Roboflow Dental Dataset

The Roboflow dental dataset [29] was additionally screened, from which:

- 56 pediatric caries images
- 8 clinically challenging healthy cases, including stainless steel crowns (metallic), zirconia crowns (tooth-colored), sealants, and amalgam restorations

**Figure 1:**
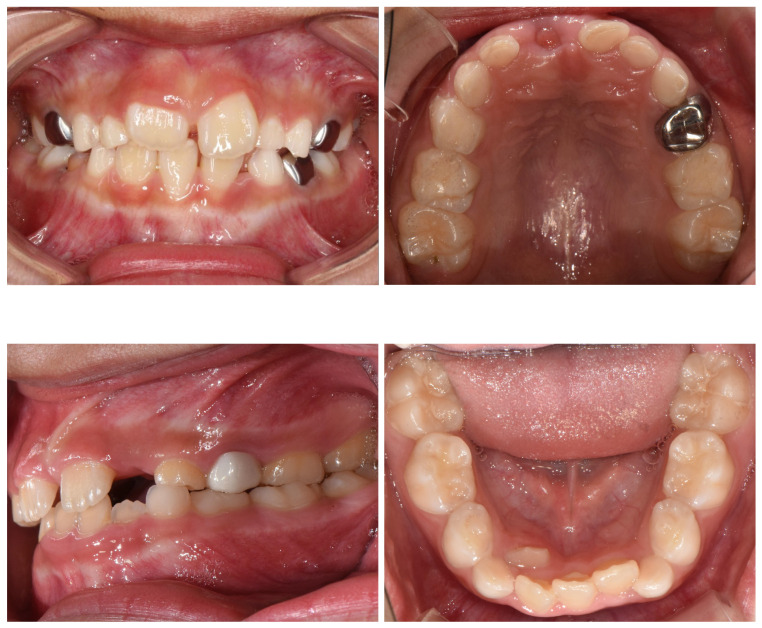
Representative examples of clinically challenging healthy cases included in the dataset. These restorations introduce reflective and textural variations that may visually resemble carious lesions, thereby reducing shortcut learning and improving model robustness.

These materials introduce diverse reflective and textural artifacts that may visually resemble carious lesions (Figure 1), thereby reducing the risk of shortcut learning and improving model robustness.

#### 3.1.2. Pediatric Filtering and Expert Validation

Because some source datasets included mixed-age populations, all candidate images underwent a two-stage screening process. First, a pediatric-only filtering protocol was applied, retaining images that demonstrated primary or mixed dentition without evidence of fully developed permanent (adult) dentition. Ambiguous cases were excluded.

Subsequently, three licensed dental experts independently reviewed the remaining images for diagnostic labeling. Each image was evaluated for the presence or absence of dental caries.

An image was included in the final dataset only if all three experts reached unanimous agreement on both pediatric eligibility and a diagnostic label (caries vs. healthy). Images with any disagreement were excluded to prioritize label certainty over dataset size and minimize annotation noise.

#### 3.1.3. Final Dataset Composition

The final curated dataset consists of 435 images (Table 2).

#### 3.1.4. Class Distribution

The final dataset distribution is as follows:

- No_Caries: 135 images (31%)
- Caries: 300 images (69%)

The dataset was divided into 70% training, 15% validation, and 15% held-out test sets while preserving class distribution. When patient identifiers were available, splitting was performed at the patient level; otherwise, images were treated as independent samples.

### 3.2. Preprocessing and Data Augmentation

The current study focuses exclusively on image-level diagnostic classification. No bounding box annotations, region-of-interest markings, or pixel-wise segmentation masks were utilized. Even when source repositories provided localization information, only global image-level labels were retained to maintain consistency across datasets.

All images were standardized to three-channel RGB format and resized to 224×224 pixels to match the input resolution required by the pretrained architectures. Pixel values were normalized using the standard ImageNet normalization parameters (mean = [0.485, 0.456, 0.406]; standard deviation = [0.229, 0.224, 0.225]) to maintain the feature distribution expected by ImageNet-initialized weights during transfer learning. Data augmentation was applied only to the training set to improve model generalization and reduce overfitting. The augmentation pipeline included random rotations (±20°), horizontal flipping, color jittering (brightness, contrast, and saturation adjustments), and mild affine transformations (translation up to 10% and scaling between 0.9 and 1.1). No augmentation was applied to the validation or test sets; only resizing and normalization were performed to ensure an unbiased evaluation of model performance.

### 3.3. Model Development

To evaluate the effectiveness of compact architectures for ECC screening, we compared three lightweight convolutional neural networks: EfficientNet-B0, ResNet-18, and MobileNetV3-Small. All models were initialized with ImageNet-pretrained weights and fine-tuned using a unified transfer learning protocol to ensure fair comparison.

For each architecture, early convolutional layers were frozen to preserve general low-level visual features while higher layers were fine-tuned for dental imagery. Specifically, we froze the EfficientNet-B0 early feature blocks, the ResNet-18 stem, the first residual block (conv1, bn1, layer1), and the first six feature blocks of MobileNetV3-Small.

The original classification layers were replaced with task-specific two-class heads. EfficientNet-B0 employed dropout (p=0.3) followed by a linear layer, ResNet-18 used a single fully connected layer, and MobileNetV3-Small retained its default lightweight classifier design, including built-in dropout.

All models were trained using identical preprocessing, augmentation, optimization, and evaluation settings to enable direct and unbiased comparison.

### 3.4. Training Procedure

To address class imbalance, we used a class-weighted cross-entropy loss. Class weights were computed from the training split only using inverse class frequency and normalized to sum to one:

(1) $$w_{c}=\frac{\frac{1}{n_{c}}}{\sum_{k}\frac{1}{n_{k}}},$$

where $n_{c}$ denotes the number of training samples in class *c*. This reweighting reduces the dominance of the majority class during optimization and encourages balanced performance across classes.

Models were optimized using Adam with an initial learning rate of $1\times 10^{-4}$. We applied a StepLR schedule that decays the learning rate by a factor of 0.1 every 10 epochs. Training was run for up to 30 epochs with a batch size of 16, and model selection was based on validation-balanced accuracy.

To mitigate overfitting, early stopping was applied on validation balanced accuracy with a patience of 7 epochs and a minimum improvement threshold of $1\times 10^{-4}$. The checkpoint achieving the highest validation balanced accuracy was retained and evaluated on the held-out test set.

Balanced accuracy was selected as the early-stopping criterion instead of standard accuracy because it accounts for class imbalance by averaging sensitivity and specificity across classes. This prevents the model from favoring the majority class and provides a more reliable indicator of clinically meaningful performance for screening tasks.

### 3.5. Implementation and Hardware

All experiments were implemented in PyTorch (v2.9.0) with CUDA 12.6 using Python 3.12 and executed on a CUDA-enabled GPU.

To ensure reproducibility, fixed random seeds were applied across all data splits, model initialization, and training procedures.

Model training, evaluation, and visualization (including ROC/PR curves, confusion matrices, and Grad-CAM explanations) were conducted within the same computational environment to guarantee consistent and comparable results across architectures.

### 3.6. Evaluation Protocol

Model performance was evaluated exclusively on the held-out test set, which remained completely unseen during training and hyperparameter selection. The model checkpoint selected for testing corresponded to the highest validation balanced accuracy to ensure unbiased model selection under class imbalance.

We report accuracy, balanced accuracy, sensitivity (recall for the Caries class), specificity, weighted F1-score, receiver operating characteristic area under the curve (ROC-AUC), and precision–recall area under the curve (PR-AUC). Balanced accuracy was prioritized as the primary performance metric because it equally weights sensitivity and specificity, providing a more reliable assessment under imbalanced class distributions. PR-AUC was additionally reported due to its greater informativeness in screening settings where positive cases are less frequent.

Model outputs are probabilistic scores in the range [0,1]. A fixed decision threshold of 0.5 was defined a priori to convert probabilities into binary predictions and ensure fair comparison across models. Sensitivity–specificity trade-offs were further analyzed using ROC and precision–recall curves.

To quantify statistical uncertainty, 95% confidence intervals for ROC-AUC and PR-AUC were estimated using non-parametric bootstrap resampling (1000 iterations). All metrics were computed at the image level. These 95% confidence intervals are interpreted as uncertainty estimates reflecting variation under patient-level sampling.

## 4. Experiments

### 4.1. Baselines

To evaluate whether compact architectures can achieve clinically meaningful performance, we benchmarked three convolutional neural networks representing different accuracy–efficiency trade-offs. Table 3 summarizes the parameter counts and computational requirements of the evaluated architectures. All models were initialized with ImageNet pretrained weights [30] and adapted via transfer learning by replacing the final classification layer with a two-class head (Caries vs. no-Caries). To mitigate overfitting on the limited medical dataset and reduce computational cost, early feature layers were frozen while deeper layers and the classification head were fine-tuned.

EfficientNet-B0: EfficientNet-B0 employs compound scaling to jointly balance network depth, width, and input resolution, achieving strong accuracy with relatively low computational cost [31]. It serves as a widely used, accuracy-oriented, lightweight baseline in medical imaging applications.

ResNet-18: ResNet-18 is a residual convolutional network that uses identity skip connections to facilitate stable gradient propagation and efficient optimization [32]. Its moderate depth and strong generalization make it a standard baseline for classification tasks.

MobileNetV3-Small: MobileNetV3-Small is designed for efficient inference on mobile and edge devices and is derived from the MobileNetV2 architecture, which introduced inverted residual blocks and linear bottlenecks for parameter-efficient feature extraction [33]. It further integrates squeeze-and-excitation modules and hardware-aware optimization to improve accuracy–latency trade-offs [13]. Owing to its low parameter count and computational footprint, the model is well-suited for deployment in community and smartphone-based screening scenarios.

MobileNetV3-Small requires less than one-quarter of the parameters of ResNet-18 and approximately half those of EfficientNet-B0 while providing substantially lower computational cost and faster inference. This makes it particularly suitable for mobile or community screening deployments.

### 4.2. Explainability

To assess model interpretability, we employed Gradient-weighted Class Activation Mapping (Grad-CAM) [34] to visualize regions contributing most strongly to the model’s predictions. Grad-CAM heatmaps were generated for representative test samples across true positive, true negative, false positive, and false negative cases to qualitatively analyze both correct detections and failure modes.

These visualizations were used solely for post hoc interpretability and were not involved in training or model selection. In general, the highlighted regions corresponded to tooth surfaces and suspected lesion areas, supporting the clinical plausibility of the learned representations.

## 5. Results

### 5.1. Quantitative Performance

ResNet-18 achieved the highest overall classification performance, obtaining the best accuracy (0.955), balanced accuracy (0.929), and weighted F1-score (0.954) (Table 4). Notably, it reached perfect sensitivity (1.00), correctly identifying all caries cases in the test set. This makes ResNet-18 particularly well-suited for screening applications where minimizing false negatives is critical. However, its specificity (0.86) was lower than that of EfficientNet-B0, indicating a higher rate of false positives.

EfficientNet-B0 demonstrated strong and well-balanced performance, achieving high sensitivity (0.93) and specificity (0.90), alongside the highest ROC-AUC (0.9778) and PR-AUC (0.9901). The superior area-under-curve metrics suggest that EfficientNet-B0 provides the strongest ranking capability and better class separability across decision thresholds, making it attractive for threshold tuning and deployment in varying clinical operating conditions.

MobileNetV3-Small achieved competitive sensitivity (0.98) but exhibited lower specificity (0.81) and balanced accuracy (0.894), reflecting a greater tendency toward false positives. Nevertheless, it maintained strong ROC-AUC (0.9524) and PR-AUC (0.9763) scores, demonstrating effective probability ranking despite reduced threshold-based performance.

Overall, these findings indicate that lightweight convolutional architectures can achieve clinically meaningful performance while offering distinct trade-offs. ResNet-18 favors maximal sensitivity, EfficientNet-B0 provides the best overall discriminative ability, and MobileNetV3-Small offers computational efficiency with competitive detection performance.

### 5.2. Confusion Matrix Analysis

**Figure 2:**
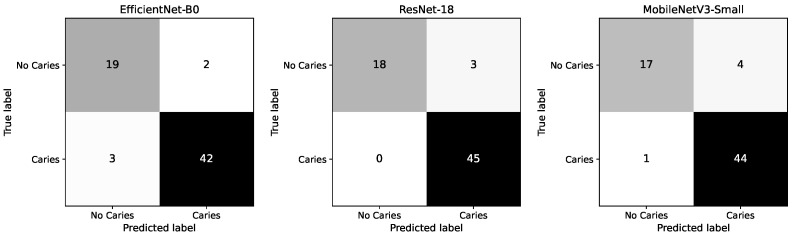
Test-set confusion matrices for the three lightweight CNN baselines. Cell values indicate the number of samples, and darker shading represents higher counts.

Figure 2 presents the confusion matrices for the three evaluated models. ResNet-18 demonstrates perfect sensitivity, correctly identifying all 45 caries cases (0 false negatives), while misclassifying 3 healthy cases as cavities. This confirms its strong suitability for screening scenarios where missing a caries is clinically undesirable.

EfficientNet-B0 achieves a balanced performance, correctly detecting 42 out of 45 caries cases (3 false negatives) and 19 out of 21 healthy cases (2 false positives), reflecting a strong trade-off between sensitivity and specificity.

MobileNetV3-Small also maintains high caries detection performance, correctly identifying 44 of 45 caries cases (1 false negative), but shows a higher number of false positives (4 healthy cases misclassified), resulting in reduced specificity compared to the other models.

Overall, the confusion matrices highlight the differing operating characteristics of the models: ResNet-18 prioritizes maximal sensitivity, EfficientNet-B0 provides the most balanced error distribution, and MobileNetV3-Small achieves competitive detection performance with slightly reduced specificity.

### 5.3. ROC/PR Curves

**Figure 3:**
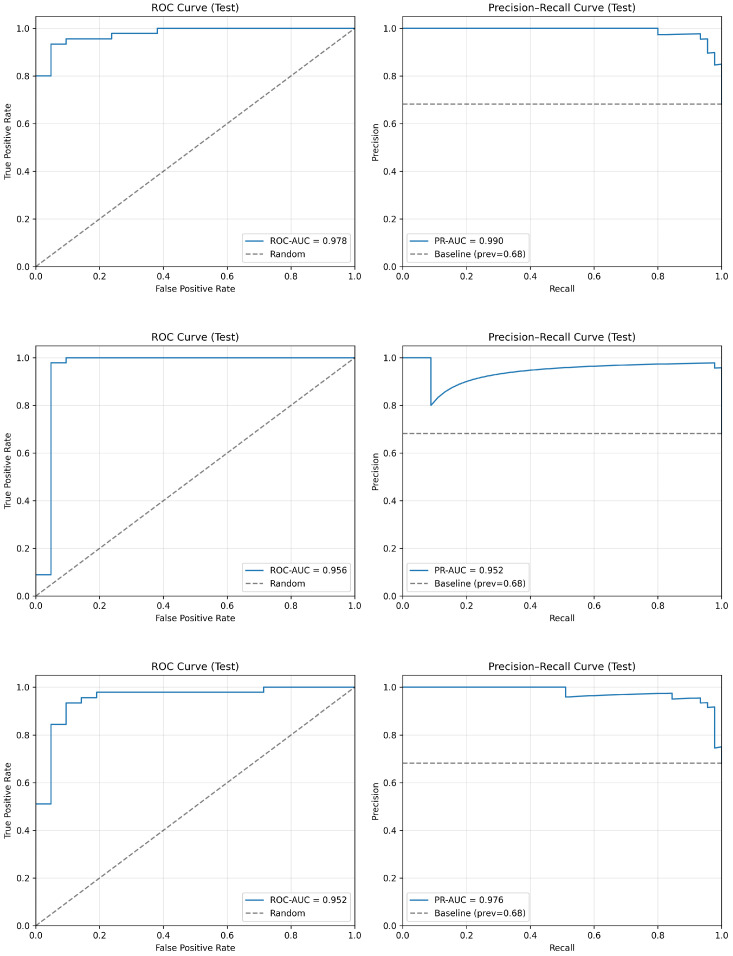
ROC and precision–recall (PR) curves on the held-out test set for the evaluated lightweight models. (Top row): EfficientNet-B0. (Middle row): ResNet-18. (Bottom row): MobileNetV3-Small. MobileNetV3-Small achieves the highest PR-AUC, while ResNet-18 shows strong balanced performance and stability across thresholds.

As shown in Figure 3, the top row (EfficientNet-B0) demonstrates the strongest overall discriminative ability, achieving the highest ROC-AUC (0.978) and PR-AUC (0.990), with curves consistently approaching the upper-left and upper-right corners of the plots. This indicates excellent class separability and precision–recall trade-off across decision thresholds.

The middle row (ResNet-18) exhibits stable and well-balanced behavior, with a high ROC-AUC (0.956) and PR-AUC (0.952). The curves show strong sensitivity across low false-positive rates, reflecting the perfect caries detection performance at the selected operating threshold.

The bottom row (MobileNetV3-Small) achieves competitive ranking performance (ROC-AUC = 0.952, PR-AUC = 0.976), maintaining high precision across moderate-to-high recall values. Although slightly below EfficientNet-B0 in overall area-under-curve metrics, it demonstrates effective probability calibration and threshold flexibility.

Overall, the ROC and PR analyses confirm that EfficientNet-B0 provides the strongest threshold-independent discrimination, while ResNet-18 emphasizes high sensitivity at the chosen operating point, and MobileNetV3-Small maintains strong ranking performance with lightweight architecture efficiency.

### 5.4. Explainability Analysis (Grad-CAM)

**Figure 4:**
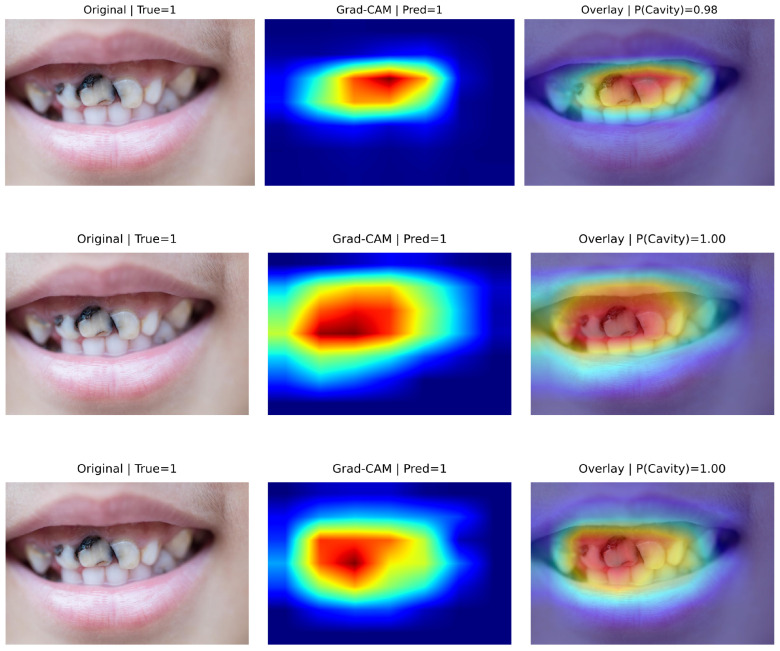
Grad-CAM visualizations for representative true positive cases (TP). (Top to bottom): EfficientNet-B0, ResNet-18, and MobileNetV3-Small. (Left): Original images. (Middle): Grad-CAM heatmaps. (Right): Overlays with predicted probabilities. In the heatmaps, warmer colors (red/yellow) indicate regions with higher contribution to the model’s prediction, while cooler colors (blue) indicate lower contribution. The model consistently attends to tooth surfaces and suspected lesion regions while largely ignoring surrounding soft tissue, indicating clinically meaningful feature localization. High prediction confidence (P(cavity) = 0.98–1.00) further supports robust detection of cavitated lesions.

Figure 4 presents representative true positive cases with Grad-CAM visualizations. In all correctly detected caries images, activation maps were predominantly concentrated over tooth structures and visually suspicious regions, including enamel surfaces and localized areas of discoloration.

Although some diffuse activation extends to adjacent oral structures in certain models, the strongest responses consistently overlap with the cavitated tooth regions. This suggests that predictions are primarily driven by clinically relevant dental features rather than background artifacts.

The corresponding prediction confidences were high (P(cavity) = 0.98–1.00), supporting stable and discriminative feature representations for cavitated lesions.

**Figure 5:**
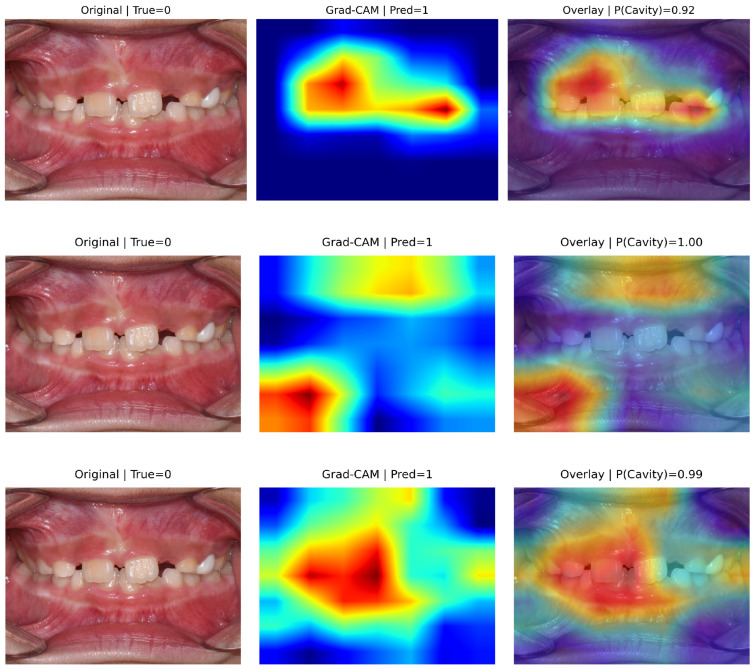
Grad-CAM visualizations for representative false positive cases (FP). (Top to bottom): EfficientNet-B0, ResNet-18, and MobileNetV3-Small. (Left): Original images. (Middle): Grad-CAM heatmaps. (Right): Overlays with predicted probabilities. In the heatmaps, warmer colors (red/yellow) indicate regions with higher contribution to the model’s prediction, while cooler colors (blue) indicate lower contribution. Although clinically labeled as non-cavitated, the models focused on visually ambiguous regions such as reflections, shadows, and enamel boundaries, which resemble lesion-like patterns. These findings illustrate typical sources of over-sensitive predictions in real-world oral photographs.

Figure 5 shows representative false-positive cases for the three models. Although these images were clinically labeled as non-cavitated, the networks predicted cavities with high confidence (P(cavity) = 0.92–1.00).

Grad-CAM visualizations indicate that the models predominantly focused on visually ambiguous dental regions, including specular reflections, enamel boundaries, and shadowed interproximal areas that resemble lesion-like intensity variations. In some cases, activation extended modestly toward adjacent gingival or soft-tissue regions; however, the strongest responses consistently overlapped with tooth structures.

These findings suggest that false positives arise primarily from challenging visual artifacts and high-contrast dental patterns rather than arbitrary background correlations. The consistently high predicted probabilities further indicate that confidence-based threshold adjustment alone may not fully mitigate such errors. Improving robustness to illumination variability, reflections, and shadow artifacts remains critical for reliable deployment in real-world oral photography settings.

**Figure 6:**
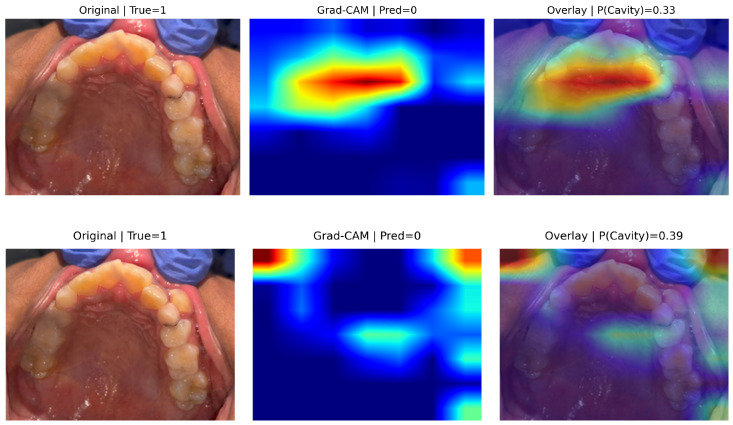
Representative false-negative examples across the two models. From (top to bottom): EfficientNet-B0 and MobileNetV3-Small (ResNet has no false negatives). In the heatmaps, warmer colors (red/yellow) indicate regions with higher contribution to the model’s prediction, while cooler colors (blue) indicate lower contribution. Although cavities were clinically present (True = 1), all models predicted the non-caries class with low caries probabilities. Grad-CAM visualizations show attention displaced from the true lesion sites toward surrounding tissue or restorations, suggesting reduced sensitivity to subtle or low-contrast cavities. Such errors are clinically critical, as missed lesions may delay early intervention.

Figure 6 presents representative false negative cases for EfficientNet-B0 (top) and MobileNetV3-Small (bottom), while ResNet-18 exhibited no false negatives. Despite clinically confirmed cavities (True = 1), both models predicted the non-caries class with relatively low caries probabilities (P(Cavity) ≈ 0.33 for EfficientNet-B0 and 0.39 for MobileNetV3-Small), indicating reduced confidence in lesion detection.

In the EfficientNet-B0 example (top row), Grad-CAM activation is concentrated primarily along broader palatal and occlusal regions rather than the localized cavitated site, suggesting misdirected spatial emphasis. In contrast, the MobileNetV3-Small example (bottom row) shows more diffuse and fragmented activation patterns, with limited overlap over the clinically relevant lesion area. This indicates reduced localization precision under challenging intraoral views.

These results suggest that false negatives arise from insufficient emphasis on small, low-contrast, or spatially localized cavities rather than arbitrary background attention. Importantly, ResNet-18 did not exhibit false negatives in the test set, consistent with its perfect sensitivity.

Given the clinical implications of missed cavities in screening settings, improving sensitivity to subtle lesion patterns—particularly under complex lighting and occlusal perspectives—remains critical. While Grad-CAM provides valuable qualitative insight into model attention behavior, rigorous quantitative evaluation of interpretability remains an open challenge [35].

## 6. Discussion

This study investigated whether compact convolutional neural networks can achieve clinically meaningful performance for automated early childhood caries (ECC) screening from intraoral photographs while remaining computationally efficient for mobile and community deployment. Overall, the results demonstrate that lightweight architectures provide strong diagnostic discrimination with high sensitivity and area-under-curve metrics, supporting the feasibility of scalable, low-resource screening systems.

### 6.1. Quantitative Performance and Model Comparison

All evaluated models achieved strong diagnostic performance, with ROC-AUC values ranging from 0.952 to 0.978 and PR-AUC values between 0.952 and 0.990 (Table 4). These results indicate excellent class separability and confirm that photographic dental images contain sufficient discriminative information for automated caries detection.

EfficientNet-B0 achieved the highest threshold-independent discrimination, obtaining the best ROC-AUC (0.978) and PR-AUC (0.990). This suggests superior ranking capability across decision thresholds and strong robustness in distinguishing caries from non-caries cases.

ResNet-18 demonstrated the strongest threshold-based classification performance, achieving the highest accuracy (0.955), balanced accuracy (0.929), and weighted F1-score (0.954), along with perfect sensitivity (1.00). This indicates that ResNet-18 reliably detected all caries cases in the test set, making it particularly well-suited for screening scenarios where minimizing false negatives is critical.

MobileNetV3-Small achieved competitive performance (ROC-AUC = 0.952, PR-AUC = 0.976) with very high sensitivity (0.98), although with reduced specificity (0.81) compared to the other models. This reflects a tendency toward over-detection, leading to more false positives but maintaining strong lesion detection capability.

Collectively, these findings demonstrate that lightweight convolutional architectures can achieve clinically meaningful performance while offering distinct trade-offs. EfficientNet-B0 provides the strongest overall discriminative ability, ResNet-18 maximizes sensitivity and balanced accuracy at the selected operating point, and MobileNetV3-Small maintains competitive detection performance with computational efficiency. These results suggest that moderate model complexity is sufficient for effective ECC detection, supporting the feasibility of deployment in mobile or resource-constrained environments.

### 6.2. Clinical Relevance of Operating Characteristics

From a screening perspective, sensitivity and PR-AUC are particularly important, as missed carious lesions may delay intervention and allow disease progression. In the present study, all models achieved high sensitivity (0.93–1.00), with ResNet-18 demonstrating perfect lesion detection on the test set. This suggests that lightweight CNNs can effectively minimize false negatives, a critical requirement in early childhood caries (ECC) screening.

EfficientNet-B0 achieved the highest PR-AUC (0.990), indicating strong precision–recall performance across operating thresholds and suggesting flexibility for threshold tuning depending on clinical context. While MobileNetV3-Small exhibited slightly lower specificity (0.81), its high sensitivity (0.98) may still be acceptable in community-based screening programs where over-referral is preferable to missed disease.

In school or primary-care settings, such systems could support non-specialist personnel by flagging potentially suspicious cases for referral, rather than replacing professional clinical examination. Nevertheless, these findings are based on retrospective image analysis and should be interpreted as decision-support evidence rather than a standalone clinical diagnosis.

### 6.3. Explainability and Model Behavior

Grad-CAM analysis provides qualitative insight into how each architecture localizes caries-related features. In true positive cases, all models predominantly concentrated activation over clinically meaningful dental structures, including enamel surfaces and visibly cavitated regions. The strongest responses consistently overlapped with lesion areas, supporting the hypothesis that the networks learned pathology-relevant visual cues rather than relying on global context alone. Although mild spatial spread toward adjacent oral structures was occasionally observed, the dominant activations remained centered on tooth regions. The high prediction confidences (P(cavity) ≈ 0.98–1.00) further indicate stable and discriminative feature representations for cavitated lesions.

False positive cases reveal characteristic failure modes. In these examples, activation maps were largely concentrated on tooth surfaces exhibiting high-contrast boundaries, reflections, enamel edges, or shadowed interproximal regions that visually resemble early lesion patterns. While some activation extended modestly toward surrounding soft tissue, the strongest responses remained within dental structures, suggesting that errors arise from visually ambiguous anatomical features rather than arbitrary background correlations. Notably, several false positives were assigned high caries probabilities (0.92–1.00), indicating that simple confidence thresholding may not fully eliminate over-detection. From a screening perspective, such conservative behavior may be clinically acceptable, as false positives typically result in additional examination rather than missed disease.

False-negative cases were observed for EfficientNet-B0 and MobileNetV3-Small, whereas ResNet-18 exhibited no false negatives in the test set. In these missed cases, predicted caries probabilities were moderate (P(cavity) ≈ 0.33–0.39), reflecting reduced confidence rather than extreme miscalibration. Grad-CAM visualizations show partial displacement of attention away from the true lesion sites, with activations concentrating on broader palatal or occlusal regions or appearing spatially diffuse. This pattern suggests diminished localization precision for subtle or low-contrast lesions, particularly under challenging intraoral perspectives. Given that missed pathology carries greater clinical risk than false alarms, improving sensitivity to small or visually subtle cavities remains an important area for future refinement.

Architectural differences were also reflected in activation behavior. EfficientNet-B0 exhibited relatively smooth and spatially coherent attention maps, consistent with its strong ranking performance (highest ROC-AUC and PR-AUC). ResNet-18 demonstrated focused lesion-centered activations aligned with its perfect sensitivity at the chosen operating point. In contrast, MobileNetV3-Small showed comparatively more fragmented and spatially dispersed attention patterns in difficult cases, which may reflect reduced representational capacity. While computationally efficient, such behavior may increase susceptibility to imaging artifacts and contribute to its lower specificity.

Overall, the interpretability findings align with the quantitative performance metrics, reinforcing the clinical plausibility of the learned representations while highlighting specific pathways for improving robustness under real-world imaging variability.

**Figure A1:**
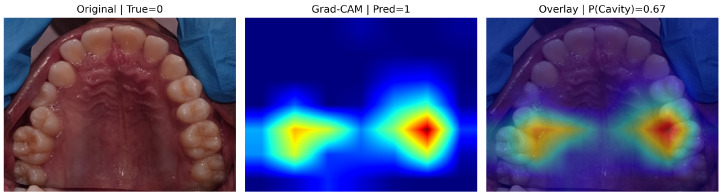
Representative false-positive Grad-CAM visualization for EfficientNet-B0. Although this image was clinically labeled as non-cavitated (True = 0), the model predicted a caries (Pred = 1) with moderate confidence (P = 0.67). The activation map highlights regions along the occlusal surfaces and enamel boundaries rather than localized pathological areas. Attention appears distributed across visually ambiguous regions, suggesting that the model may rely on texture and structural cues that resemble early lesion patterns, leading to over-detection.

**Figure A2:**
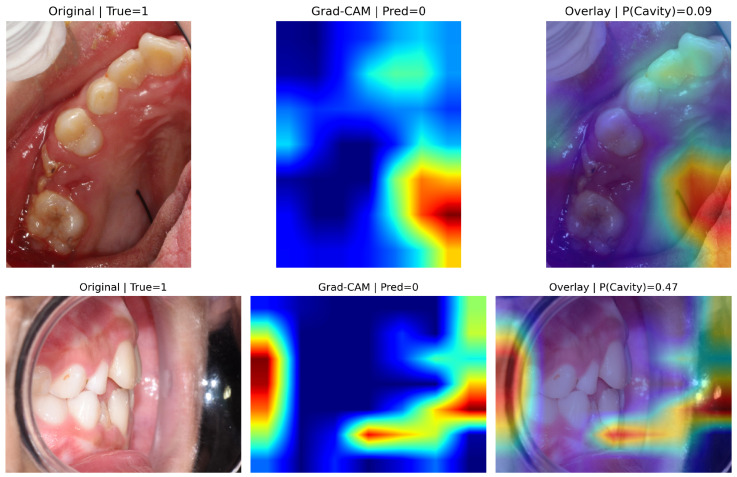
Representative false-negative Grad-CAM visualizations for EfficientNet-B0. Although these images were clinically labeled as cavitated (True = 1), the model predicted the non-caries class (Pred = 0) with low caries probabilities (P = 0.09–0.47). The activation maps demonstrate weak, diffuse, or mislocalized attention, frequently concentrated on peripheral regions, reflections, or adjacent enamel structures rather than the clinically confirmed lesion areas. This pattern indicates insufficient localization of pathological features and reduced sensitivity.

**Figure A3:**
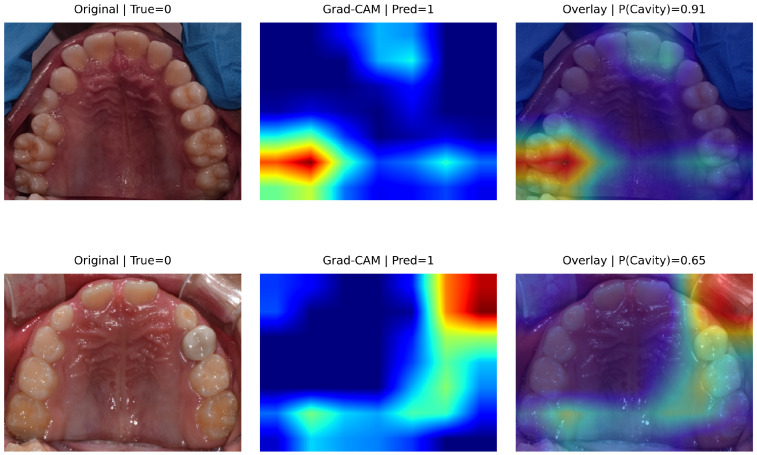
Representative false-positive Grad-CAM visualizations for ResNet-18. Although these images were clinically labeled as non-cavitated (True = 0), the model predicted cavities (Pred = 1) with moderate-to-high confidence (P(Cavity) = 0.65–0.91). Grad-CAM maps show broad and diffuse activations spanning large occlusal regions rather than focusing on localized pathological areas. Attention appears distributed across enamel surfaces and structural contours, suggesting reliance on global texture cues and leading to reduced specificity.

**Figure A4:**
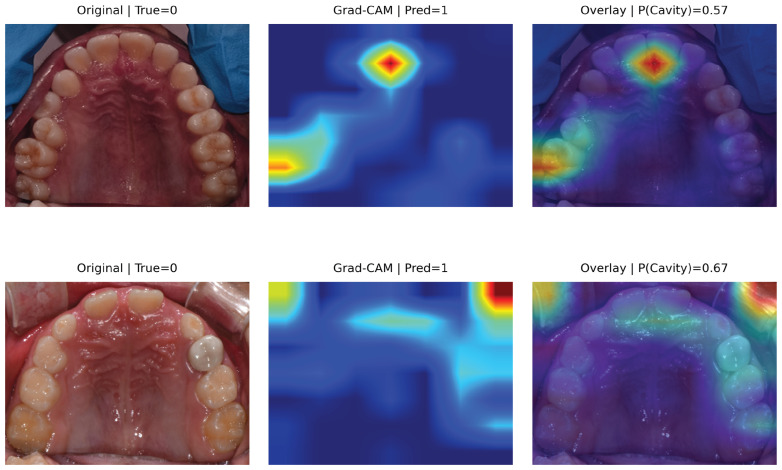
Representative false-positive Grad-CAM visualizations for MobileNetV3-Small. Although these images were clinically labeled as non-cavitated (True = 0), the model predicted cavities (Pred = 1) with moderate confidence (P(Cavity) = 0.57–0.67). Grad-CAM maps show scattered and fragmented activations across multiple tooth regions rather than focused attention on localized lesion-like areas. This dispersed activation pattern suggests sensitivity to subtle texture variations or illumination artifacts, contributing to reduced specificity.

**Figure A5:**
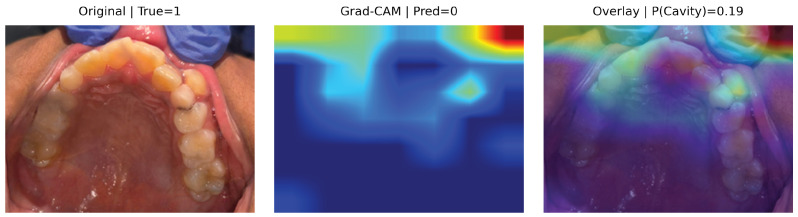
Representative false-negative Grad-CAM visualization for MobileNetV3-Small. Although this image contains a clinically confirmed cavitated lesion (True = 1), the model predicted the non-caries class (Pred = 0) with low confidence (P(Cavity) = 0.19). The Grad-CAM map shows weak and spatially diffuse activations that fail to concentrate on the lesion region, instead highlighting peripheral enamel and surrounding structures. This mislocalized attention suggests insufficient pathological feature emphasis and reduced sensitivity to localized cavitation.

Additional qualitative Grad-CAM failure cases for each model are presented in Appendix A (Figure A1, Figure A2, Figure A3, Figure A4 and Figure A5).

In several false-positive cases, Grad-CAM activations extend beyond tooth surfaces and partially overlap with surrounding contextual regions, including areas near dental instruments (e.g., retractors or mirrors), as visible in the second rows of Figure A3 and Figure A4. This behavior suggests a potential contextual bias, where the models may rely on acquisition-related cues rather than lesion-specific enamel features. Such patterns are consistent with shortcut learning [36], in which models exploit non-pathological correlations in the data [37]. Future work may mitigate this effect through artifact-aware preprocessing or region-of-interest constraints focused on tooth structures.

### 6.4. Efficiency and Deployability

A key contribution of this work is demonstrating that mobile-friendly architectures such as MobileNetV3-Small can achieve clinically meaningful performance while maintaining substantially lower computational complexity than larger models. Although its specificity was slightly reduced compared to EfficientNet-B0 and ResNet-18, MobileNetV3-Small maintained high sensitivity (0.98) and strong ranking performance (ROC-AUC = 0.952; PR-AUC = 0.976), supporting its viability for screening-oriented applications.

Such efficiency is critical for real-world deployment, particularly on smartphones, tablets, or edge devices, where memory, latency, and energy constraints limit heavier architectures. The results therefore suggest that compact CNNs offer a favorable accuracy–efficiency trade-off and represent practical, deployment-ready baselines for teledentistry and community screening programs.

### 6.5. Ethical Considerations for Pediatric AI Screening

The proposed system is intended solely as a screening decision-support tool and not a replacement for professional dental diagnosis. Because the target population is pediatric, human clinical oversight is necessary to prevent false reassurance or unnecessary concern. The model should only assist referral decisions, and all suspected cases must be confirmed by a qualified dentist. In addition, the use of anonymized images and privacy protection is essential when handling pediatric data. Prospective clinical validation is required before real-world clinical deployment.

### 6.6. Limitations

Several limitations should be considered. First, annotations were image-level rather than pixel-level, preventing quantitative assessment of lesion localization accuracy. Second, Grad-CAM provides qualitative interpretability but does not constitute a formal causal explanation of model decisions, and standardized evaluation metrics for explainability remain limited. Third, the binary classification setting does not capture lesion severity, staging, or progression, which are clinically relevant for treatment planning. Finally, the study was conducted on a retrospective dataset, and prospective validation in real-world screening environments is required to confirm generalizability. Model performance may vary across populations with different disease prevalence. Therefore, the system should be interpreted as a decision-support screening aid, and threshold calibration may be required before real-world deployment.

In summary, this study demonstrates that lightweight deep learning models can achieve clinically meaningful performance for ECC detection from oral photographs while maintaining computational efficiency suitable for large-scale deployment. EfficientNet-B0 achieved the strongest threshold-independent discrimination, ResNet-18 demonstrated perfect sensitivity at the selected operating point, and MobileNetV3-Small provided competitive performance with reduced computational complexity. These findings support the feasibility of deploying compact CNN architectures for automated dental screening in resource-constrained and community settings.

## 7. Conclusions

Early childhood caries (ECC) remains one of the most prevalent chronic diseases in pediatric populations, and timely identification is critical for preventing pain, infection, and long-term oral health complications. Scalable screening solutions are particularly needed in community and resource-constrained settings, where access to specialist dental care is limited.

In this work, we investigated whether compact convolutional neural networks can provide clinically meaningful performance for automated caries detection from intraoral photographs. Using a patient-level evaluation protocol, class-imbalance–aware training, and clinically relevant metrics with bootstrap confidence intervals, we benchmarked three architectures—EfficientNet-B0, ResNet-18, and MobileNetV3-Small. Overall, the findings demonstrate strong screening performance for dental caries detection using pediatric oral photographs. EfficientNet-B0 achieved the strongest threshold-independent discrimination, obtaining the highest ROC-AUC and PR-AUC, while ResNet-18 demonstrated the best threshold-based operating performance, including the highest balanced accuracy and perfect sensitivity at the selected operating point. MobileNetV3-Small maintained competitive detection capability with substantially lower computational complexity, supporting its suitability for deployment in resource-constrained environments.

Qualitative Grad-CAM analysis further supported the clinical plausibility of the learned representations, showing attention predominantly concentrated on anatomically meaningful tooth regions rather than irrelevant background cues. Error analysis revealed interpretable failure modes: false positives were frequently associated with reflections, shadows, and high-contrast enamel boundaries, whereas false negatives involved subtle or low-contrast lesions with displaced or diffuse activation patterns. These findings highlight opportunities for improved robustness to illumination variability, better calibration, and enhanced sensitivity to early-stage pathology.

Overall, the results suggest that lightweight deep learning models constitute effective and deployment-ready baselines for ECC screening from photographs and may enable accessible decision-support tools for teledentistry and community health programs. However, this study does not constitute a clinical validation trial, and the models should not be interpreted as diagnostic devices. Broader external validation on multi-center datasets and prospective workflow studies will be required before real-world clinical adoption. Future work will explore robustness across imaging conditions and devices, lesion localization refinement, and integration into mobile health platforms to support practical field deployment.

## Figures and Tables

**Table 1:** Comparison of the proposed approach with representative prior dental caries detection studies.

Study	Model	Dataset	Performance
[11]	CNN	3000 radiographic	Accuracy 82–89%, AUC 0.917
[23]	DL system with object detection models	1000 smartphone intraorals	Avg. Recall 56.2%
[12]	U-Net variant	1500 Panoramic X-ray	Accuracy 95%
[22]	U-Net CNN	304 bitewing radiographs	Precision 63.29%, Recall 65.02%, F1-score 64.14%
Proposed Study	MobileNetV3-Small, ResNet-18, EfficientNet-B0	435 intraorals photographs	ROC-AUC 0.943, PR-AUC 0.985

**Table 2:** Dataset Composition by Source.

Source	No_Caries	Caries	Total
Mendeley Dataset	127	30	157
AAP Gallery	0	14	14
OMNI	0	120	120
Kaggle	0	80	80
Roboflow	8	56	64
Total	135	300	435

**Table 3:** Model complexity comparison. Parameter count, approximate FLOPs.

Model	Params (M)	FLOPs (G)@24×24
MobileNetV3-Small	2.5	0.06
EfficientNet-B0	5.3	0.39
ResNet-18	11.7	1.8

**Table 4:** Test-set performance of lightweight CNN baselines.

Model	Acc.	Bal. Acc.	F1 (w)	Sens.	Spec.	ROC-AUC	PR-AUC
EfficientNet-B0	0.924	0.919	0.925	0.930	0.900	0.978	0.990
ResNet-18	0.955	0.929	0.954	100.000	0.860	0.956	0.952
MobileNetV3-S	0.924	0.894	0.920	0.980	0.810	0.952	0.976

Acc. = Accuracy; Bal. Acc. = Balanced Accuracy; F1(w) = Weighted F1; Sens. = Sensitivity; Spec. = Specificity.

## Data Availability

The datasets used in this study are publicly available. The sources of the datasets are described in the Materials section of the manuscript. Additional processed data may be available from the corresponding author upon reasonable request.
